# Ethnobotanical, Phytochemical, Pharmacological, and Toxicological Aspects of *Persicaria hydropiper* (L.) Delarbre

**DOI:** 10.1155/2014/782830

**Published:** 2014-04-16

**Authors:** A. K. M. Moyeenul Huq, Jamia Azdina Jamal, Johnson Stanslas

**Affiliations:** ^1^Centre for Drug and Herbal Research, Faculty of Pharmacy, Universiti Kebangsaan Malaysia, Jalan Raja Muda Abdul Aziz, 50300 Kuala Lumpur, Malaysia; ^2^Pharmacotherapeutics Unit, Department of Medicine, Faculty of Medicine and Health Sciences, Universiti Putra Malaysia, 43400 Serdang, Selangor Darul Ehsan, Malaysia

## Abstract

*Persicaria hydropiper* (L.) Delarbre, belonging to Polygonaceae family, is a common weed found in most of the temperate countries including Bangladesh, China, Malaysia, and Japan. The plant is also referred to as “marsh pepper” or “smart weed.” It appears to be a useful herb with evidence-based medicinal properties. The present work addresses the botanical description, traditional uses, phytochemistry, pharmacology, and toxicology of *P. hydropiper*. All plant parts have been commonly used in the traditional systems of medicines. Flavonoids are the major group of phytochemical components followed by drimane-type sesquiterpenes and sesquiterpenoids, as well as phenylpropanoids. Different extracts and plant parts showed remarkable pharmacological activities including antioxidant, antibacterial, antifungal, antihelminth, antifeedant, cytotoxicity, anti-inflammatory, antinociceptive, oestrogenicity, antifertility, antiadipogenicity, and neuroprotection. Mutagenicity and acute and subchronic toxicities of the plant were also reported. *P. hydropiper* has tremendous medicinal properties that could further be investigated for the development of evidence-based herbal products.

## 1. Plant Botanical Description and Distribution

*Persicaria hydropiper* (L.) Delarbre belongs to the family of Polygonaceae. The synonyms for this species include* Persicaria hydropiper* (L.) Spach,* P. hydropiper* (L.) Opiz,* P. hydropiper* (L.) H. Gross,* Polygonum hydropiper *L., and* P. hydropiper* var.* projectum* Stanford [1–4]_._ The species is commonly known as marsh-pepper smartweed, marsh-pepper knotweed, smartweed, or water pepper [5–7] and is also called la liao in China [8], bishkatali or pakarmul in Bangladesh [9], and daun senahun in Malaysia [10].

Based on the information stated in the Flora of North America and Flora of China [4, 8],* P. hydropiper* is an annual plant of 40–70 cm tall. Briefly, it has a decumbent to ascending or erect branched and glabrous stem. The leaves are lanceolate or elliptic-lanceolate (4–10 × 0.4–2.5 cm) and glabrous with petiole (0.1–0.8 cm), cuneate base, acute to acuminate apex, ciliated margin, and sessile attachment with stipule. The terminal and axillary inflorescences (0.3–18.0 × 0.5–0.9 cm) are either erect or nodding with glabrous peduncle (0.1–0.5 cm), ascending pedicels, and 3–5 flowers. The flowers have greenish proximal and white or pink distal perianth, obovate tepals, 6–8 stamens, and 2-3 styles.

*P. hydropiper* is distributed worldwide and found native in temperate and tropical Asia including Western Asia, Caucasus, Siberia, Middle Asia, Russian Far East, China, Eastern Asia, Indian Subcontinent, Indo-China, and Malesia; Europe region such as Northern Europe, Middle Europe, East Europe, Southeastern Europe, and Southwestern Europe; Northern Africa and Australia [11]. The plant generally grows in wet areas at watersides and in marshes [12] and is usually predominant in agricultural fields [13]. It is also commonly distributed to highland sites with highly organic, moist, or silty areas [14].

## 2. Traditional Uses

*Persicaria hydropiper* has a strong peppery taste and is commonly used as a hot-tasting spice, food flavor, and garnish for a variety of traditional dishes [15–17]. The Japanese people use the young shoot as spice and garnish with raw fish such as “sashimi” for its pungent taste [18], while the water or ethanol leaf extract served as a food additive to preserve pickles, dressing, and cooked foods [19]. In Southeast Asia, the Chinese and Malays use the leaves in traditional laksa dishes [16].

Most importantly,* P. hydropiper* also has a wide range of traditional uses for medicinal purposes. In Europe, the plant has been used as diuretic and emmenagogue [20] and to regulate menstrual irregularities [21]. In addition, decoction of the whole plant, either alone or mixed with other medicinal plants, is also given for diarrhea, dyspepsia, itching skin, excessive menstrual bleeding, and hemorrhoids [22]. The leaves and seeds are used in a folk medicine against cancer [23]. The Romanian people in Oltenia utilized infusion of the aerial part as astringent and cicatrising, as well as for gastric, pulmonary problems, and uterine hemorrhages [24]. The use of bruised leaves and seeds as vesicants has also been reported [25].

In India, the Mishing women in Assam take the dried root powder of* P. hydropiper* for termination of pregnancy and it may lead to permanent sterility if taken continuously for more than a year [26, 27]. Leaf's juice is consumed for uterine disorders [28]. In Arunachal Pradesh, the whole plant extract and ground plant paste are used as fish poisons [28, 29], whereas the leaf infusion is used to relieve colic pain [30]. The plant has also been utilized as natural dyes [31].

In Bangladesh, the Garo tribe uses the leaf juice for menstrual pain, the leaf paste to stop bleeding, and the whole plant as pesticide for stored grains [32]. Another tribe of Tripura uses the mixture of crushed* P. hydropiper* leaf with black pepper for headache [33]. In a district of Sylhet, the crushed plant helps to arrest hemorrhage and in Rema-Kalenga, the leaves are used for stomach pain [34]. The leaf juice has been given for treating many health problems like headache, pain, toothache, liver enlargement, gastric ulcer, dysentery, loss of appetite, and dysmenorrhea, while the roots are used as stimulant and their juice is applied to wounds, skin diseases, and painful carbuncles [35].

In Vietnam, the stems and leaves are taken for snake-bite and as diuretic and anthelmintic [12]. In China, the plant is consumed to prevent ovulation and cease pregnancy [36], while the root is used as stimulant, diuretic, carminative, tonic, and anthelmintic [37].

This plant has been found to be toxic to pigs and sheep [38].

## 3. Phytochemical Constituents

*P. hydropiper* has been reported to contain mainly flavonoids, sesquiterpenes, sesquiterpenoids, and phenylpropanoids (Table 1).

**Figure 1:**
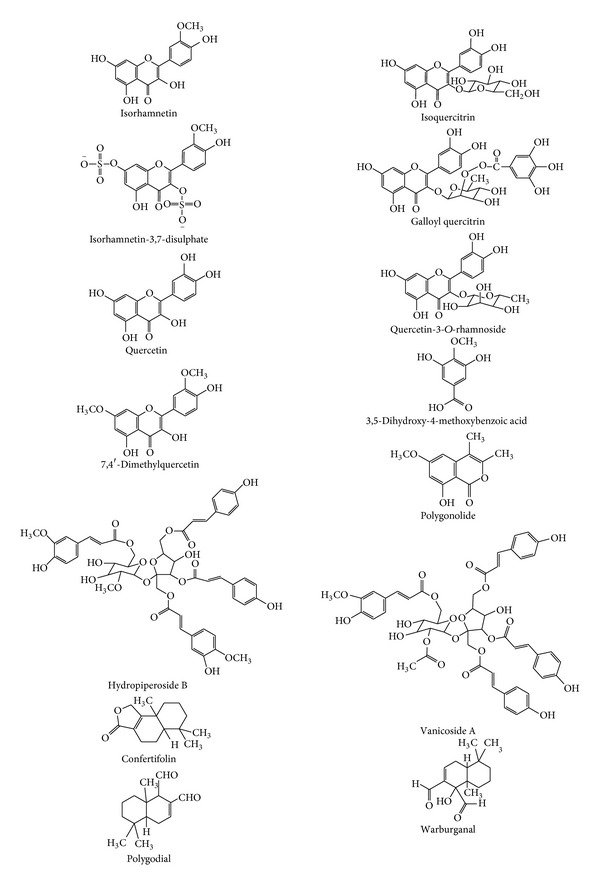
Bioactive phenolics and sesquiterpenoids of* Persicaria hydropiper*.

Various extracts and fractions of* P. hydropiper* whole plant and herbs were found to contain flavonoids, such as (+)-catechin, (−)-epicatechin, hyperin, isoquercitrin (Figure 1), isorhamnetin (Figure 1), kaempferol, quercetin (Figure 1), quercitrin, rhamnazin and rutin [39–41]; drimane-typed sesquiterpenes, such as 3-*β*-angeloyloxy-7-epifutronolide, 7-ketoisodrimenin, changweikangic acid A, dendocarbin L, (+)-fuegin, futronolide, polygonumate, and (+)-winterin [42]; phenylpropanoid esters, including hydropiperosides A and B (Figure 1), and vanicosides A (Figure 1), B and E [43]; as well as phenolic acids, such as caffeic acid, chlorogenic acid and *ρ*-coumaric acid [41].

Methanol (80–100%) extract of the leaves had mainly flavonoids, including apigenin-7-*O*-glucoside, galloyl kaempferol-3-*O*-glucoside, galloyl quercetin-3-*O*-glucoside, galloyl quercetin-3-*O*-rhamnoside, galloyl quercitrin (Figure 1), isoquercitrin, isoquercitrin-3-glucoside, isorhamnetin, isorhamnetin-3,7-disulphate (Figure 1), kaempferol rutinoside, kaempferol-3-*O*-glucoside, percicarin/persicarin, quercitrin, quercetin (Figure 1), quercetin-3-*O*-glucoside, quercetin-3-*O*-*β*-D-glucuronide, quercetin-3-*O*-rhamnoside (Figure 1), quercetin-3-sulphate, rhamnazin, rhamnazin-3-sulphate, rhamnetin, scutillarein, tamarixetin-3-*O*-*β*-glucoside-7-sulphate, 3′-methylquercetin, 6-hydroxyluteolin, 6-hydroxyluteolin-7-*O*-*β*-D-glucopyranoside, 6-hydroxyapigenin, and 7,4′-dimethylquercetin (Figure 1) [16, 17, 44–46]; phenylpropanoids, such as hydropiperosides and vanicosides A, B, and D; and 3,5-dihydroxy-4-methoxybenzoic acid (Figure 1) [17]. Additionally, the leaf diethyl ether extract was found to have sesquiterpenes and sesquiterpenoids, such as confertifolin (Figure 1), drimenol, (+)-fuegin, isodrimeninol, isopolygodial, isopolygonal, polygodial (Figure 1), polygodial acetal, polygonal, polygonic acid, polygonone, valdiviolide, warburganal (Figure 1), and 11-ethoxycinnamolide [18, 47], whereas the essential oil had acetic acid, confertifolin, diethyleneglycol monoacetate, ethyl benzene, ethyl propionate, and n-propyl acetate [48].

The methanol extract of stems and leaves was reported to contain hydropiperosides A and B and vanicosides A, B, and E [43], while the petroleum extract of top part had polygodial [49] and the methanol extract of roots had anthraquinone, ellagic acid 3,3′-di-*O*-methyl ether, gallic acid, hydropiperosides, and polygonolide (Figure 1) [50, 51]. Several sesquiterpenes and sesquiterpenoids, such as confertifolin, drimenol, isodrimeninol, isopolygodial, polygodial, polygonal and warburganal, have been isolated from diethyl ether extract of the seeds [18, 52]. (+)-catechin, (+)-epicatechin, and (+)-epicatechin-3-O-gallate were produced by the callus and suspension-cultured cells of* P. hydropiper* [53], whereas polygodial was detected in the shoot cultures [54].

## 4. Pharmacological Properties

Several reports on pharmacological properties of* P. hydropiper* are available to support the ethnomedicinal uses of the plant including antioxidant, antibacterial, antifungal, antihelminth, antifeedant, cytotoxicity, anti-inflammatory, antinociceptive, oestrogenicity, anti-fertility, anti-adipogenicity, anticholinesterase, and neuroprotection. Toxicological effects of* P. hydropiper* are also described.

### 4.1. Antioxidant Activity

Flavonoids are powerful antioxidants that can protect the human body from free radicals [55]. Isoquercitrin and 7, 4′-dimethylquercetin isolated from the methanol extract of* P. hydropiper* leaves were found to inhibit lipid peroxidation using ferric thiocyanate (FTC) method with ID_50_ of 0.6 and 1.5 ppm, respectively [44]. Yagi et al. [46] also studied the antioxidant activity of quercetin-3-sulphate, isorhamnetin-3,7-disulphate, and tamarixetin-3-glucoside-7-sulphate isolated from the methanol leaf extract. Amongst the sulphated flavonoids, isorhamnetin-3,7-disulphate gave the strongest inhibition against lipid peroxidation even compared to *α*-tocopherol and quercetin and the formation of superoxide anion and xanthine oxidase, compared to quercetin.

Peng et al. [16] investigated the antioxidant properties of 10 flavonoids isolated from the leaves of* P. hydropiper*, that is, quercitrin, kaempferol-3-glucoside, 6-hydroxyapigenin, galloyl kaempferol-3-glucoside, scutillarein, 6-hydroxyluteolin, 6-hydroxyluteolin 7-*O*-*β*-D-glucopyranoside, quercetin 3-*O*-*β*-D-glucuronide, galloyl quercitrin, and quercetin, showing Trolox equivalent antioxidant capacity (TEAC) values of 1.39–6.14 against 2,2′-azinobis(3-ethyl-benzothiazoline-6-sulphonic acid (ABTS) radicals. Galloyl quercitrin was the most powerful antioxidant found in the study (TEAC = 6.14) compared to quercitrin (TEAC = 3.46) and its aglycone, quercetin (TEAC = 4.65).

Hydropiperoides B and vanicoside A isolated from the* P. hydropiper* methanol leaf extract demonstrated antioxidant activity in 1,1-diphenyl-2-picrylhydrazyl (DPPH) free radical-scavenging assay with half maximal scavenging concentration (SC_50_) values of 23.4 and 26.7 *μ*g/mL, respectively, compared to ascorbic acid (SC_50_ 22.0 *μ*g/mL) [43]. Noor Hashim et al. [17] also reported the antioxidant activity of ethyl acetate fraction of methanol leaf extract against DPPH free radicals with IC_50_ value of 13.30 *μ*g/mL, whereby the 3,5-dihydroxy-4-methoxybenzoic acid (IC_50_ 8.08 *μ*g/mL), quercetin (IC_50_ 11.14 *μ*g/mL), and quercetin-3-*O*-rhamnoside (IC_50_ 18.46 *μ*g/mL) were found to be most active as compared to vitamin C (IC_50_ 6.80 *μ*g/mL).

### 4.2. Antibacterial Activity

Confertifolin isolated from the leaf essential oil of* P. hydropiper* showed strong/good antibacterial activity against* Enterococcus faecalis *(MIC 31.25 *μ*g/mL) as compared to a positive standard, streptomycin (MIC 25 *μ*g/mL), but did not inhibit the growth of* Bacillus subtilis, Erwinia* sp.,* Escherichia coli*,* Klebsiella pneumoniae*,* Pseudomonas aeruginosa*,* Staphylococcus aureus*, and* S. epidermidis *[48]. On the other hand, Kubo et al. [56] revealed that polygodial had moderate bactericidal action against* Bacillus subtilis* (minimum bactericidal concentration, MBC 100 *μ*g/mL),* Staphylococcus aureus* (MBC 100 *μ*g/mL),* Escherichia coli* (MBC 100 *μ*g/mL), and* Salmonella choleraesuis* (MBC 50 *μ*g/mL).

### 4.3. Antifungal Activity

Confertifolin isolated from the leaf essential oil was also found to have potent antifungal activity against* Epidermophyton floccosum*,* Curvularia lunata, *and* Scopulariopsis* sp. (MIC 7.81 *μ*g/mL) and moderate activity against* Aspergillus niger*,* Botrytis cinerea*,* Magnaporthe grisea*,* Trichophyton mentagrophytes*,* Trichophyton rubrum* (MTCC 296 and clinical isolate) and* Trichophyton simii* (MIC 16.62–125 *μ*g/mL) as compared to fluconazole (MIC < 12.5–100 *μ*g/mL) and ketoconazole (MIC < 12.5 *μ*g/mL) [48].

Polygodial was also reported to inhibit* Candida albicans*,* C. utilis*,* C. krusei*,* Cryptococcus neoformans*,* Saccharomyces cerevisiae*,* Epidermophyton floccosum*,* Trichophyton mentagrophytes*,* T. rubrum*, and* Penicillium marneffei* [57, 58]. It showed potent fungicidal activity against* C. albicans* [58]. In another studies, polygodial isolated from* Warburgia* species and* P. hydropiper* showed fungicidal activity against* S. cerevisiae* [59, 60], via several mechanisms such as decreasing cytoplasmic and mitochondrial glutathione and increasing production of reactive oxygen species [61] and inhibition of mitochondrial ATPase [62, 63]. Later, Fujita and Kubo [64] revealed that polygodial, a nonionic surfactant, denatured the lipid-protein conformation of the cell membrane and interacted with L-cysteine containing cytoplasmic materials such as glutathione.

### 4.4. Anthelmintic Activity

Methanol (99%) extract of* P. hydropiper *aerial plant part (50 mg/mL) displayed anthelmintic activity against adult earthworms,* Pheretima posthuma*,* in vitro* with time of paralysis and death of 12.44 and 18.19 min, respectively, compared to the positive standard, piperazine citrate (10 mg/mL, time of paralysis = 24.00 min, time of death = 38.00 min) [65].

### 4.5. Antifeedant Activity

Hot water extract of* P. hydropiper* leaves (10% w/v) was significantly effective against the bean aphids,* Aphis craccivora, *with 87.6–94.5% mortality (*P* < 0.01) 7 days after the application of spray at 227 L/ha [14].

Warburganal was previously reported to have strong antifeedant activity against African armyworms,* Spodoptera exempta* [66] and aphids [67]. Polygodial was found to be active antifeedant against a variety of aphids (*Aphis craccivora*,* Myzus persicae*, and* Rhopalosiphum padi*), African or Egyptian cotton leafworm (*Spodoptera littoralis*), and whiteflies (*Bemisia tabaci*) [67–70].

### 4.6. Cytotoxic Activity

Various fractions of* P. hydropiper* herb and root methanol extracts were tested for antiproliferative activity against cervical epithelial adenocarcinoma (HeLa), skin epidermoid carcinoma (A431), and breast epithelial adenocarcinoma (MCF7) cells; only hexane fraction of the root methanol extract (30 *μ*g/mL) was found to inhibit HeLa cell proliferation (54.75% inhibition) [71]. Polygodial exhibited cytotoxicity against Ehrlich ascites tumor cells and mouse lymphocytic leukemia-derived L1210 cells [72].

An* in vivo* study performed by Raihan et al. [65] showed that the methanol (99%) extract of* P. hydropiper* aerial part had antiproliferative activity against Ehrlich Ascites Carcinoma (EAC) cells inoculated intraperitoneally (i.p.) in Swiss-Webster albino male mice. The extract at a dose of 50 mg/kg/day (i.p.) significantly (*P* < 0.001) inhibited (84.54%) EAC cell growth, decreased tumor weight to 7.85 g, and improved mean survival time (68.0% increase of life span) of EAC bearing mice, as compared to the positive standard, bleomycin (0.3 mg/kg, i.p.) with values of 98.55%, 7.05 g, and 94.66%, respectively.

### 4.7. Anti-inflammatory Activity

Methanol (99%) leaf extract of* P. hydropiper* inhibited production of inflammatory mediators* in vitro* such as nitric oxide (NO), tumor necrosis factor (TNF)-*α*, and prostaglandin (PG) E_2_ in lipopolysaccharide-induced RAW264.7 cells and peritoneal macrophages by suppressing the activation of Src/Syk/NF-kB and IRAK/AP-1/CREB pathways [73].

Furuta et al. [51] demonstrated the anti-inflammatory property of polygonolide isolated from methanol extract of* P. hydropiper* root by inhibiting reversed passive Arthus reaction. A dose of 100 mg/kg polygonolide administered orally 1 hour before induction of inflammation on the rat skin was able to inhibit 39.2% (*P* < 0.05) of the acute inflammation.

Polygodial, a major compound previously isolated from the barks of* Drimys winteri*, was reported to be responsible for inhibiting guinea-pig ileum and tracheal contractility* in vitro* induced by several mediators associated with asthmatic and allergic responses, including acetylcholine, histamine, bradykinin, KCl, 9,11-dideoxy-9*α*, 11*α*-methano-epoxy prostaglandin F_2*α*_, substance P, and tachykinin NK_2_ receptor [74]. It also showed inhibitory effect of ovalbumin-sensitized and compound 48/80-stimulated contraction of guinea-pig trachea [74]. da Cunha et al. [75] revealed that polygodial had anti-inflammatory and antiallergic properties* in vivo* via various mechanisms of actions including inhibition of mice paw oedema induced by prostaglandin E_2_ (ID_50_ 107 *μ*mol/kg at 180 min), bradykinin (ID_50_ 86 *μ*mol/kg at 60 min), substance P (ID_50_ 83 *μ*mol/kg at 180 min), dextran (ID_50_ 17 *μ*mol/kg at 60 min), platelet activating factor (58% inhibition at 60 min), carrageenan (ID_50_ 32 *μ*mol/kg), and ovalbumin (80% inhibition at 240 min); mice ear oedema was stimulated by arachidonic acid (ID_50_ 141.3 *μ*mol/kg), capsaicin (ID_50_ 169 *μ*mol/kg), and croton oil (44% inhibition), pleurisy was induced by substance P and histamine, and anaphylactic shock was stimulated by ovalbumin.

### 4.8. Antinociceptive Activity

Ethyl acetate extract of* P. hydropiper* whole plant exhibited significant dose-dependent antinociceptive activity in Swiss albino mice (42.86% inhibition at 250 mg/kg and 54.95% at 500 mg/kg, *P* < 0.001) as compared to aminopyrine (73.62% at 50 mg/kg) by acetic acid-induced writhing method, suggesting its analgesic potential [76].

Mendes et al. [77] isolated polygodial from the barks of* D. winteri* and found that it (0.1 to 10 mg/kg, administered by intraperitoneal injection) was able to inhibit mice abdominal contractions induced by acetic acid (ID_50_ 0.8 mg/kg), zymosan (ID_50_ 2.1 mg/kg,) and kaolin (ID_50_ 2.6 mg/kg). Polygodial also demonstrated distinct systemic, spinal, and supraspinal antinociceptive effect on mice, mainly preventing the formalin- and capsaicin-induced neurogenic pain, via several mechanisms including binding to the k and d subtypes of opioid receptors, activation of pertussis toxin-sensitive Gi/Go-protein, binding to *α*_1_-adrenoceptors and serotoninergic system [78]. Neurogenic antinociceptive and thermal antihyperalgesic effects were observed in neonatal treatment of rats [79].

### 4.9. Oestrogenic and Antifertility Activity

Garg et al. [80] first reported the antifertility activity of ethanol extract of* P. hydropiper* root on female albino rats. Recently, the methanol root extract administered orally to ovary-intact and ovariectomized adult albino rats at a dose of 1000 mg/kg body weight/day for three consecutive oestrous cycles (12 days) was found to induce endometrial proliferation and follicular growth that was evidenced by the regulation of endometrial protein expression, suggesting its oestrogenic property comparable to estradiol-17*β* [26, 81]. Further investigations have demonstrated that the steroid-containing fraction of* P. hydropiper* methanol root extract, administered subcutaneously at a dose of 5 mg/kg/day, stimulated proliferation of uterine epithelium of ovariectomized adult albino rats [27]. The fraction also stimulated expression of various uterine proteins in ovary intact (molecular weight *≈*150000, *≈*90000, *≈*82000, *≈*56000, *≈*43000, and *≈*38000) and ovariectomized (*≈*38000) rats but reduced expression of proteins (*≈*65000 and *≈*38000) in pregnant rats of 5-6 days after implantation [82]. The latter was indicated by the suppressed expression of estrogen-sensitive transforming growth factor-*β*I in the primary decidual zone of the implantation sites during day 6 of gestation, suggesting the antifertility activity [83].

### 4.10. Antiadipogenic Activity

Methanol extract of* P. hydropiper* whole plant (1 *μ*g/mL) and its flavonol components, isoquercitrin (50 *μ*M) and isorhamnetin (50 *μ*M), were shown to activate the Wnt/*β*-catenin signaling in HEK 293 cells containing pTOPFlash reporter gene, increase nuclear localization of *β*-catenin in 3T3-L1 adipocyte cells, and inhibit adipocyte differentiation, suggesting its potential application as antiobesity agents and for associated disorders [84].

### 4.11. Anticholinesterase Activity

Cholinesterase assays using acetylcholinesterase and butyrylcholinesterase enzymes were conducted on* P. hydropiper*. However, the methanol extract, fractions (hexane, dichloromethane, ethyl acetate, butanol, and aqueous) and changweikangic acid A did not exhibit anticholinesterase activity [17, 42].

### 4.12. Neuroprotective Activity

Persicarin was discovered as a component of the* P. hydropiper* methanol leaf extract [45]. As a matter of fact, Ma et al. [85] reported that persicarin isolated from the stems and leaves of* Oenanthe javanica* demonstrated significant neuroprotective activity (40.8–74.5% protection at 10.0 *μ*M, *P* < 0.001) in glutamate-induced neurotoxicity of rat cortical cells by inhibition of intracellular calcium influx, intracellular nitric oxide production, and cellular peroxide formation, as well as by increasing the antioxidant activities of superoxide dismutase, glutathione reductase, and glutathione peroxidase. Depending on the amount of persicarin in* P. hydropiper*, its extract could potentially possess neuroprotective activity.

## 5. Toxicology

Kuroiwa et al. [86] stated that the* P. hydropiper* ethanol leaf fraction containing 7.0% polygodial gave positive mutagenicity in two tests, that is, the Ames test using* Salmonella typhimurium* TA 100 and TA 98 and the chromosomal aberrations using Chinese hamster-derived CHL/IU cells, but it was negative for micronuclei in mouse bone marrow cells. It was also previously reported that polygodial was negatively mutagenic in the Ames test using TA 100, TA 98, and TA 2637 strains of* S. typhimurium* [71] and in the mammalian cell V79/HGPRT assay [87].

Acute toxicity in Swiss-Webster albino male mice was conducted by Raihan et al. [65], in which methanol (99%) extract of* P. hydropiper* aerial part (20–600 mg/kg) was injected intraperitoneally. After 24 hours, no mortality was observed up to 400 mg/kg, but 100% mice died at 600 mg/kg, suggesting the LD_50_ of the extract to be 500 mg/kg (i.p.).

Kuroiwa et al. [86] also investigated the subchronic toxicity of WPE in male and female F344/DuCrj rats given ad libitum for 13 weeks. The no observed-adverse effect was found with 1000 ppm ethanol leaf fraction containing 7.0% polygodial (57.4 and 62.9 mg/kg/day for males and females, resp.), whereby there were no obvious clinical signs and no significant changes in food consumption, hematology and serum biochemistry, body and organ weights, and histopathology of organs of the tested rats.

The aerial parts cause blister of the skin upon repeated handling that could be due to the skin irritant polygodial [49, 88]. Polygodial isolated from the bark of* D. winteri* was found to increase extracellular glutamate concentrations via concurrently inhibiting glutamate uptake by rat astrocytes and slices of cortex, striatum, and hippocampus and increasing glutamate release by synaptosomes, suggesting possible neurotoxic effect of polygodial [89].

## 6. Conclusions

The numerous ethnobotanical uses of* P. hydropiper* had drawn the attention of the scientists to investigate its traditional claims. The above discussion clearly gives us a perception on the scientific evidence of different pharmacological properties of this species which supports a number of its traditional uses such as antibacterial, antifungal, antihelminth, antifeedant, anticancer, anti-inflammatory, antinociceptive, oestrogenicity and antifertility uses. The antiallergic, antiadipogenic, and neuroprotective properties of the plant provided new knowledge on the extension of its uses. The plant contained several remarkable pharmacologically active compounds; for example, polygodial was found to have antibacterial, antifungal, antifeedant, anti-inflammatory, antinociceptive, and antiallergic properties; polygonolide had anti-inflammatory activity. Warburganal acted as antifeedant; confertifolin had antibacterial and antifungal activities; persicarin demonstrated neuroprotective activity; isoquercitrin, quercetin, quercetin-3-*O*-rhamnoside, 7,4′-dimethylquercetin, galloyl quercitrin, isorhamnetin-3,7-disulphate, 3,5-dihydroxy-4-methoxybenzoic acid, hydropiperoside B, and vanicoside A showed antioxidant properties, while isoquercitrin and isorhamnetin were antiadipogenic. Oral consumption of ethanol leaf fraction containing 7% polygodial was found to be safe* in vivo*. Thus, this plant serves as a promising candidate as a multipurpose herbal medicinal agent owing to its economical viability and being a reservoir of many significant medicinal properties in treating diseases and ailments related to microbial infections, inflammation, pain, allergy, uterine disorders, fertility, obesity, and improvement of memory.

## Figures and Tables

**Table 1:** List of chemical constituents isolated from *Persicaria hydropiper*.

Class of compound	Name of compound	Plant part	Type of extract	References
Flavonoids	Apigenin-7-*O*-glucoside	Leaves	MeOH	[17]
	(+)-Catechin	Whole plant	MeOH (80%)	[40]
		Herbs	MeOH fraction	[41]
		Callus and suspension-cultured cells	Acetone	[53]
	(−)-Epicatechin	Whole plant	MeOH (80%)	[40]
		Herbs	MeOH fraction	[41]
		Callus and suspension-cultured cells	Acetone	[53]
	(−)-Epicatechin-3-*O*-gallate	Callus and suspension-cultured cells	Acetone	[53]
	Galloyl quercitrin	Leaves	MeOH (80%)	[16]
	Galloyl quercetin-3-*O*-glucoside	Leaves	MeOH	[17]
	Galloyl kaempferol-3-*O*-glucoside	Leaves	MeOH	[17]
			MeOH (80%)	[16]
	Galloyl quercetin-3-*O*-rhamnoside	Leaves	MeOH	[17]
	Hyperin	Whole plant	MeOH (80%)	[40]
	Isoquercitrin	Whole plant	MeOH (80%)	[40]
		Leaves	MeOH	[44]
	Isoquercitrin 3-glucoside	Leaves	MeOH	[45]
	Isorhamnetin	Whole plant	MeOH (80%)	[40]
		Herbs	EtOAc fraction	[39]
		Leaves	MeOH	[45]
	Isorhamnetin 3,7-disulphate	Leaves	MeOH	[46]
	Kaempferol	Whole plant	MeOH (80%)	[40]
		Herbs	EtOAc fraction	[39]
			MeOH fraction	[41]
	Kaempferol rutinoside	Leaves	MeOH	[17]
	Kaempferol-3-*O*-glucoside	Leaves	MeOH	[17]
			MeOH (80%)	[16]
	Percicarin/persicarin	Leaves	MeOH	[45]
	Quercitrin	Whole plant	MeOH (80%)	[40]
		Leaves	MeOH (80%)	[16]
	Quercetin	Whole plant	MeOH (80%)	[40]
		Herbs	EtOAc fraction	[39]
			MeOH fraction	[41]
		Leaves	MeOH	[17, 44, 45]
			MeOH (80%)	[16]
	Quercetin 3-sulphate	Leaves	MeOH	[46]
	Quercetin-3-*O*-glucoside	Leaves	MeOH	[17]
	Quercetin-3-*O*-rhamnoside	Leaves	MeOH	[17]
	Quercetin-3-*O*-*β*-D-glucuronide	Leaves	MeOH (80%)	[16]
	Rhamnazin	Herbs	EtOAc	[39]
		Leaves	MeOH	[45]
	Rhamnazin-3-sulphate	Leaves	MeOH	[45]
	Rhamnetin	Leaves	MeOH	[17]
	Rutin	Whole plant	MeOH (80%)	[40]
		Herbs	MeOH fraction	[41]
	Scutellarein	Leaves	MeOH (80%)	[16]
	Tamarixetin 3-*O*-*β*-glucoside-7-sulphate	Leaves	MeOH	[46]
	3′-Methylquercetin	Leaves	MeOH	[44]
	6-Hydroxyluteolin	Leaves	MeOH (80%)	[16]
	6-Hydroxyluteolin-7-*O*-*β*-D-glucopyranoside	Leaves	MeOH (80%)	[16]
	6-Hydroxyapigenin	Leaves	MeOH (80%)	[16]
	7,4′-Dimethylquercetin	Leaves	MeOH	[44]
Phenylpropanoids	Hydropiperoides	Roots	MeOH	[50]
		Leaves	MeOH	[17]
	Hydropiperoides A	Stems and leaves	MeOH	[43]
	Hydropiperoides B	Stems and leaves	MeOH	[43]
	Vanicoside A	Stems and leaves	MeOH	[43]
		Leaves	MeOH	[17]
	Vanicoside B	Stems and leaves	MeOH	[43]
		Leaves	MeOH	[17]
	Vanicoside D	Leaves	MeOH	[17]
	Vanicoside E	Stems and leaves	MeOH	[43]

Sesquiterpenes and sesquiterpenoids	Changweikangic acid A	Whole plant	MeOH	[42]
	Confertifolin	Leaves	Diethyl ether	[18]
			Essential oil	[48]
		Seeds	Diethyl ether	[18, 52]
	Dendocarbin L	Whole plant	MeOH	[42]
	Drimenol	Leaves	Diethyl ether	[18]
		Seeds	Diethyl ether	[18]
	(+)-Fuegin	Whole plant	MeOH	[42]
		Leaves	Diethyl ether	[47]
	Futronolide	Whole plant	MeOH	[42]
	Isodrimeninol	Leaves	Diethyl ether	[18]
		Seeds	Diethyl ether	[18, 52]
	Isopolygodial	Leaves	Diethyl ether	[18]
		Seeds	Diethyl ether	[18, 52]
	Isopolygonal	Leaves	Diethyl ether	[47]
	Polygodial	Top part	Petroleum	[49]
		Leaves	Diethyl ether	[18]
		Seeds	Diethyl ether	[18, 52]
		Shoot cultures	—	[54]
	Polygodial acetal	Leaves	Diethyl ether	[47]
	Polygonumate	Whole plant	MeOH	[42]
	Polygonal	Leaves	Diethyl ether	[18]
		Seeds	Diethyl ether	[18, 52]
	Polygonic acid	Leaves	Diethyl ether	[47]
	Polygonone	Leaves	Diethyl ether	[47]
	Valdiviolide	Leaves	Diethyl ether	[47]
	Warburganal	Leaves	Diethyl ether	[18]
		Seeds	Diethyl ether	[18]
	(+)-Winterin	Whole plant	MeOH	[42]
	3-*β*-Angeloyloxy-7-epifutronolide	Whole plant	MeOH	[42]
	7-Ketoisodrimenin	Whole plant	MeOH	[42]
	11-Ethoxycinnamolide	Leaves	Diethyl ether	[47]

Others	Acetic acid	Leaves	Essential oil	[48]
	Anthraquinone	Roots	MeOH	[50]
	Caffeic acid	Herbs	MeOH fraction	[41]
	Chlorogenic acid	Herbs	MeOH fraction	[41]
	*ρ*-Coumaric acid	Herbs	MeOH fraction	[41]
	Diethylene glycol monoacetate	Leaves	Essential oil	[48]
	Ellagic acid 3,3′-di-*O*-methyl ether	Roots	MeOH	[50]
	Ethyl benzene	Leaves	Essential oil	[48]
	Ethyl propionate (propionic acid, ethyl ester)	Leaves	Essential oil	[48]
	Gallic acid	Roots	MeOH	[50]
	3,5-Dihydroxy-4-methoxybenzoic acid	Leaves	MeOH	[17]
	Polygonolide	Roots	MeOH	[51]
	n-Propyl acetate	Leaves	Essential oil	[48]
